# Therapeutic Approaches to Genetic Ion Channelopathies and Perspectives in Drug Discovery

**DOI:** 10.3389/fphar.2016.00121

**Published:** 2016-05-10

**Authors:** Paola Imbrici, Antonella Liantonio, Giulia M. Camerino, Michela De Bellis, Claudia Camerino, Antonietta Mele, Arcangela Giustino, Sabata Pierno, Annamaria De Luca, Domenico Tricarico, Jean-Francois Desaphy, Diana Conte

**Affiliations:** ^1^Department of Pharmacy – Drug Sciences, University of Bari “Aldo Moro”Bari, Italy; ^2^Department of Basic Medical Sciences, Neurosciences and Sense Organs, University of Bari “Aldo Moro”Bari, Italy; ^3^Department of Biomedical Sciences and Human Oncology, University of Bari “Aldo Moro”Bari, Italy

**Keywords:** ion channels pharmacology, channelopathies, physiopathology, drug discovery and development, genetics

## Abstract

In the human genome more than 400 genes encode ion channels, which are transmembrane proteins mediating ion fluxes across membranes. Being expressed in all cell types, they are involved in almost all physiological processes, including sense perception, neurotransmission, muscle contraction, secretion, immune response, cell proliferation, and differentiation. Due to the widespread tissue distribution of ion channels and their physiological functions, mutations in genes encoding ion channel subunits, or their interacting proteins, are responsible for inherited ion channelopathies. These diseases can range from common to very rare disorders and their severity can be mild, disabling, or life-threatening. In spite of this, ion channels are the primary target of only about 5% of the marketed drugs suggesting their potential in drug discovery. The current review summarizes the therapeutic management of the principal ion channelopathies of central and peripheral nervous system, heart, kidney, bone, skeletal muscle and pancreas, resulting from mutations in calcium, sodium, potassium, and chloride ion channels. For most channelopathies the therapy is mainly empirical and symptomatic, often limited by lack of efficacy and tolerability for a significant number of patients. Other channelopathies can exploit ion channel targeted drugs, such as marketed sodium channel blockers. Developing new and more specific therapeutic approaches is therefore required. To this aim, a major advancement in the pharmacotherapy of channelopathies has been the discovery that ion channel mutations lead to change in biophysics that can in turn specifically modify the sensitivity to drugs: this opens the way to a pharmacogenetics strategy, allowing the development of a personalized therapy with increased efficacy and reduced side effects. In addition, the identification of disease modifiers in ion channelopathies appears an alternative strategy to discover novel druggable targets.

## Introduction

Ion channels are membrane proteins that selectively regulate ion fluxes across the membranes of cells and cellular organelles, their gating mechanism depending on changes in membrane voltage, ligand binding or physical and chemical stimuli. The presence of distinct ion channel isoforms and their age-dependent and tissue-specific expression allow the fine regulation of many cellular functions, such as cell excitability, contraction, neurotransmitter and hormone release, gene expression, ion and water homeostasis (Hibino et al., 2010; Jan and Jan, 2012; Catterall and Swanson, 2015; Jentsch, 2015; Zamponi et al., 2015).

**FIGURE 1:**
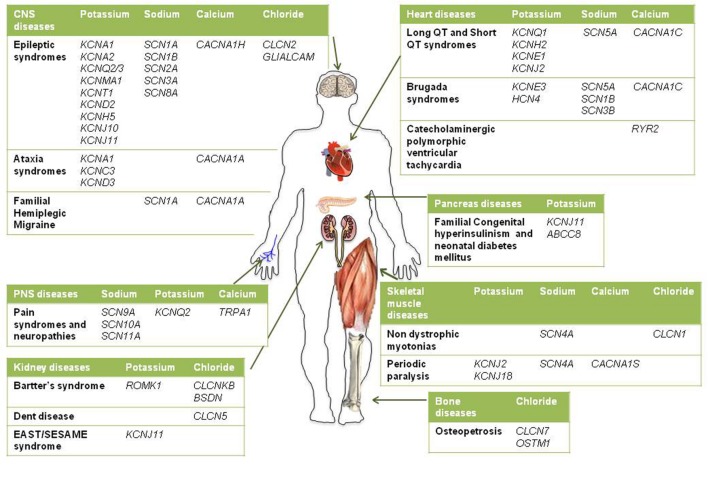
**Schematic diagram illustrating the main inherited ion channelopathies of CNS, PNS, skeletal muscle, heart, kidney, pancreas, and bone**.

Given the pivotal roles played by ion channels and their extensive distribution, it is not surprising that mutations in ion channels genes, or their interacting proteins, cause specific inherited ion channelopathies, ranging from relatively common ones, such as idiopathic epilepsies, to very rare diseases (**Figure 1**). Despite the differences in their genetic origin and clinical setting, ion channelopathies share some common features regarding pathophysiology and therapeutic approach. In general, loss- or gain-of-function mutations translate into a principal “organ-specific” clinical phenotype (Cannon, 2015; Spillane et al., 2016). The clinical manifestation of the disease depends on the type of mutation and functional consequences on channel activity, the cellular and subcellular localization of the channel isoform, and the channel oligomeric assembly (Kullmann and Waxmann, 2010). Since first discovery, the clinical spectrum of most channelopathies has broadened as new carrier families and new mutations have been identified. In some cases, the range of symptoms associated with specific channelopathies are not easily explained at a molecular and functional level and, besides the disease-causing mutation in a particular ion channel gene, they might stem from the interplay of different genes and life conditions acting as disease modifiers (Kullmann and Waxmann, 2010; Kearney, 2011; Furby et al., 2014; Swaggart and McNally, 2014; Portaro et al., 2015). Other channelopathies are further complicated by comorbidity, including seizures, myotonia, neurodevelopmental delay, arrhythmias, or diabetes that led to the definition of multiorgan syndromes. As a consequence of such genetic and clinical complexity and of the rarity of affected patients, a precise diagnosis for several channelopathies is still difficult to be made and a genotype-phenotype correlation cannot be easily defined. This, in turn, can subtsantially affect the choice of the most appropriate therapeutic management. The elevated cost of drug discovery restrains the development of new drugs for these rare diseases that are generally neglected by pharmaceutical companies. Furthermore, as patients’ groups are very small, clinical trials of statistical significance are difficult to run. A few channelopathies, such as sodium channel myotonias (SCMs) or neonatal diabetes, may benefit from a targeted pharmacotherapy (Desaphy et al., 2013b; De Franco et al., 2015). In some cases, a pharmacogenetic approach have been explored successfully (Yang et al., 2012; Desaphy et al., 2013b). Yet, most of inherited channelopathies lack specific treatments, and medications for these disorders are often empiric and symptomatic, acting, for instance, to restore membrane excitability or counteract inflammation and pain (Spillane et al., 2016). Notoriously, these drugs are marginally effective and may work inconsistently in different patients.

Today, about 5% of the available marketed drugs are small molecules able to modulate ion channel activity in a number of diseases. Some of these compounds are also enrolled in the clinical management of channelopathies. Na_v_ channels blockers have been successfully employed for decades as anticonvulsants, antiarrhythmics, and local anesthetics (Catterall and Swanson, 2015). Great interest is emerging from the possibility to develop sodium channel inhibitors for the treatment of neuropathic pain and to repurpose marketed blockers for other medical indications (Desaphy et al., 2013a, 2014; Matthews and Hanna, 2014; de Lera Ruiz and Kraus, 2015). KCNQ openers have been placed in clinics for their anticonvulsant effects (Humphries and Dart, 2015) and KATP channels are promptly targeted by sulfonylureas in neonatal diabetes (De Franco et al., 2015). Calcium channels blockers are widely used as symptomatic antiarrhythmics. Conversely, chloride channels represent a relatively under-explored target class for drug discovery (Verkman and Galietta, 2009; Imbrici et al., 2015a; Jentsch, 2015). Unfortunately, several drugs targeting ion channels, such as sodium channel modulators, are not able to discriminate among ion channel isoforms, hence at risk of causing unwanted side effects (Catterall, 2014; Grunnet et al., 2014). With a few exceptions, much-needed specific ion channels modulators are lacking.

Therefore, to guarantee patients suffering from channel opathies a personalized therapy, novel molecules are under development and pharmacological strategies have been explored to identify novel drug targets. In principle, the better understanding of the genetic and functional defects underlying channelopathies will allow to deliver patients a precision medicine specifically targeting the biological effects of the disease-causing mutation, such as a biophysical or folding defect. Drug design and development are being greatly assisted by the recently identified crystal structures of several ion channels, by computer modeling, by high-throughput screening methods, by novel genetic studies and, finally, by transgenic and pharmacologically induced cell and animal models of channelopathies (Feng et al., 2010; Hibino et al., 2010; Jan and Jan, 2012; Tikhonov and Zhorov, 2012; Paşca et al., 2014; Jentsch, 2015; Rubinstein et al., 2015; Zamponi et al., 2015; Zou, 2015). Here we summarize the main pharmacological strategies performed to treat major channelopathies caused by mutations in voltage-gated and inward rectifying ion channels. Although, the involvement of other classes of ion channels and transporters, including neurotransmitter-gated channels (Spillane et al., 2016), cyclic nucleotide-gated channels, volume-regulated and calcium-activated anion channels (Hoffmann et al., 2015) is also well-established in channelopathies, these are not detailed in this review. Finally, although rare, some channelopathies may serve as a useful paradigm for understanding and treatment of more common multifactorial disorders.

## Molecular Mechanisms of Ion Channelopathies

Inherited channelopathies result from loss or gain of function mutations in genes coding for ion channels or their accessory subunits. Genetic variants include small deletions, insertions, frame-shifts, stop codons, missense, splice-site mutations and, recently, also exon deletions or duplications have been reported (Catterall, 2014; Imbrici et al., 2015b; Spillane et al., 2016). The majority of inherited mutations alter ion channel biophysical properties, such as voltage-dependent gating, kinetics, single channel conductance, ion selectivity, or modulation by signaling pathways. Some mutations reduce channel surface expression as a consequence of altered synthesis, defective folding and trafficking to the membrane, or increased degradation (Bechi et al., 2015; Mall and Galietta, 2015). In general, loss-of-function mutations in K^++^ or Cl^-^ channels or gain-of-function mutations in Ca^2+^ or Na^+^ channels lead to hyperexcitability disorders, such as epilepsy or myotonia. However, gain-of-function mutations of neuronal K^+^ channels or loss-of-function mutations of Na^+^ channels that decrease excitability in inhibitory interneurons can lead to epilepsy as well. Thus, the final effect of a mutation depends on the specific expression pattern, exact stochiometry and functional role of the ion channels involved. This knowledge is fundamental to design appropriate therapies.

## Central Nervous System Channelopathies

### Epilepsy

Epilepsy is a brain disorder due to abnormal synchronous discharges of neuronal networks in the brain that causes convulsions, muscle spasms, and often loss of consciousness (Spillane et al., 2016). A broad spectrum of epileptic syndromes exists, often comorbid with other clinical features, such as autistic traits and other psychiatric conditions and with cardiac arrhythmias (Splawski et al., 2004; Sicca et al., 2011; Catterall, 2014; Guglielmi et al., 2014). Different classes of ion channels play critical roles in maintaining the balance between excitatory and inhibitory inputs in the brain, thus it is no surprise that gain or loss of function mutations in their genes are associated to epilepsy.

Diverse voltage gated sodium channel subtypes, ensuring action potential generation and propagation in the CNS (Na_v_1.1, Na_v_1.2, Na_v_1.3, and Na_v_1.6) have been associated with multiple seizure disorders (Catterall, 2014). More than 300 mutations in *SCN1A* (Na_v_1.1) are responsible for genetic epilepsy syndromes ranging in severity from simple febrile seizures to Generalized Epilepsy with Febrile Seizures (GEFS+), an autosomal dominant epilepsy disorder associated to missense mutations, to Severe Myoclonic Epilepsy of Infancy (SMEI or Dravet syndrome; prevalence: <1/40,000), the most severe form of often intractable epilepsy mainly caused by truncation or deletion mutations in Nav1.1 channels (Catterall, 2014). Dominant mutations in *SCN2A* encoding Na_v_1.2 channels cause Benign Familial Neonatal-Infantile Seizures (BFNIS; prevalence: <1/1000,000), a mild seizure syndrome that responds positively to treatment with anti-epileptic drugs (AEDs), and generally remits by 1 year of age (Berkovic et al., 2004). In addition, *de novo* mutations in Na_v_1.2 can cause more severe phenotypes including developmental delay and intractable seizures (Ogiwara et al., 2009; Liao et al., 2010). Other epileptic phenotypes have been associated with mutations in *SCN3A* (Na_v_1.3), *SCN8A* (Na_v_1.6), and in *SCN1B* (Na_v_1.1 β subunits; Vanoye et al., 2013; Larsen et al., 2015; O’Malley and Isom, 2015; Wagnon and Meisler, 2015). Electrophysiological and behavioral studies on mouse models of SMEI and GEFS+ indicated that progressive loss of Nav1.1 activity in inhibitory GABAergic interneurons may cause reduced GABA release and network hyperexcitability in different brain regions, resulting in seizure syndromes of increasing severity (Tang et al., 2009; Catterall, 2014; Rubinstein et al., 2015). Beside epilepsy, impairment in GABAergic interneurons also contributes to autistic-like behavior, hyperactivity and cognitive impairment observed in SMEI patients (Bender et al., 2012; Rubinstein et al., 2015).

Voltage-dependent potassium channels concur to action potential repolarization and resting membrane potential, set the firing frequency of neurons and dampen abnormal excitatory inputs. Moreover, inward rectifier potassium channels contribute to the maintenance of resting potential and to the transport and buffering of K^+^ across membranes. Thus, disruption of K^+^ channels in specific brain areas is often associated with increased susceptibility to seizures (for reviews see D’Adamo et al., 2015b; Miceli et al., 2015a). Patients affected by episodic ataxia type 1, a form of episodic ataxia with myokymia (see below) caused by loss-of-function mutations in *KCN1A* (coding for K_v_1.1 channel), often report abnormal EEGs, and several animal models carrying *kcna1* gene defects show an increased susceptibility to seizures (Smart et al., 1998). Loss- and gain-of-function mutations in *KCNA2* (encoding the potassium channel K_v_1.2) have been identified in patients with epileptic encephalopathy, intellectual disability, delayed speech development and sometimes ataxia (Syrbe et al., 2015). Loss-of-function mutations in *KCNQ2* or *KCNQ3* (encoding for K_v_7.2 and K_v_7.3 channels) cause Benign Familial Neonatal Convulsions (BFNC; prevalence: 100 reported families), a form of juvenile epilepsy characterized by tonic or clonic episodes spontaneously disappearing during the first year of life (Miceli et al., 2015a). In the brain, heteromeric channels composed of K_v_7.2 and K_v_7.3 subunits underlie the M-current that regulates neuronal excitability in the sub-threshold range for action potential generation and limits repetitive firing (Soldovieri et al., 2011). A decrease in M-current amplitude by 25% is sufficient to cause neonatal epilepsy of variable clinical severity depending on the extent of K^+^ channel impairment caused by the specific mutation. Remarkably, *de novo* gain-of-function mutations in K_v_7.2 channels have been found in neonates affected by early-onset epileptic encephalopathy presenting with pharmacoresistant seizures and various degrees of developmental delay (Miceli et al., 2015b). In this case, the gain of potassium current in inhibitory interneurons likely increases the excitability of hippocampal CA1 pyramidal neurons leading, in turn, to epilepsy. Recently, several gain of function mutations in *KCNT1* gene (coding for Kca4.1, a sodium-activated potassium channel also named Slo2.2 or SLACK) have been identified in patients with two different types of epilepsy occurring in infancy or childhood: Malignant Migrating Partial Seizures of Infancy (MMPSI; also called Epilepsy of Infancy with Migrating Focal Seizures, EIMFS; prevalence: <1/1000,000) and Autosomal Dominant Nocturnal Frontal Lobe Epilepsy (ADNFLE; prevalence: over 100 reported families; Lim et al., 2016; Tang et al., 2016). In these patients, intellectual disability and psychiatric features have also been observed. Gain-of-function mutations in the *KCNJ10* gene (coding for the K_ir_4.1 potassium channel) have been reported in children with autistic traits, seizures and intellectual disability (Sicca et al., 2011; Guglielmi et al., 2014). Other genes of potassium channel and their accessory subunits have been implicated in complex epileptic phenotypes, including *KCNV2* (K_v_8.2), *KCNMA1* (BK), *KCNH5* (K_v_10.2), *KCNC1* gene (K_v_3.1), *KCND2* (K_v_4.2; Guglielmi et al., 2014; D’Adamo et al., 2015b; Miceli et al., 2015a; Spillane et al., 2016).

Some studies reported polymorphisms or mutations in the chloride channel genes *CLCN2* (ClC-2) and *CLCN1* (ClC-1) in epileptic patients but the involvement of these channels in epilepsy remains controversial (Saint-Martin et al., 2009; Niemeyer et al., 2010; Chen et al., 2013). One study also showed the unexpected location of ClC-1 mRNA transcripts and protein in several mouse and human brain areas, opening new perspectives to the role of the “skeletal muscle” ClC-1 channels in regulating brain excitability and susceptibility to seizures (Chen et al., 2013). Defects in ClC-2, in the astrocytic membrane protein MLC1 and in the adhesion molecule GlialCAM, underlie human leukoencephalopathies, degenerative disorders affecting the white matter of the brain and associated with myelin and astrocyte vacuolization (Depienne et al., 2013; Hoegg-Beiler et al., 2014). Mutations in both MLC1 and GlialCAM, have been associated with rare megalencephalic leukoencephalopathy with subcortical cysts (MLC1 and 2; prevalence: <1/1000,000), characterized by macrocephaly, developmental delay and seizures that appears in the first years of life (López-Hernández et al., 2011). It has been recently shown that GlialCAM, MLC1 and ClC-2 interact *in vivo*, with GlialCAM targeting both MLC1 and ClC-2 to astrocytes junctions and increasing ClC-2 chloride current, and MLC1 also affecting the localization of ClC-2 and GlialCAM in the glia (Hoegg-Beiler et al., 2014; Brignone et al., 2015). Despite the pathophysiology of MLC remains unclear, studies from different genetic animal models suggest that reduction or change in ClC-2 currents may at least in part account for the impaired glial ion homeostasis in both MLC forms (Blanz et al., 2007).

Gain of function mutations in the calcium channel gene *CACNA1C* (coding for Ca_v_1.2 channel), is associated with Timothy syndrome (TS; prevalence: <1/1000,000), a rare multiorgan system disorder that comprises long QT syndrome, seizures, autistic traits, dysmorphic features, developmental delay and immunodeficiency (Splawski et al., 2004; Bader et al., 2011; Diep and Seaver, 2015). Children die prematurely mainly for cardiac ventricular fibrillation (Heyes et al., 2015). Gain-of-function mutations in *CACNA1H* (Ca_v_3.2) channels have been found in various forms of idiopathic generalized epilepsies, such as absence epilepsy (Eckle et al., 2014; Wang et al., 2015). Studies from an animal model carrying one Ca_v_3.2 mutation associated with childhood absence epilepsy suggest that Ca_v_3.2 mutant channels potentiate NMDA-mediated synaptic transmission via increasing local Ca^2+^ influx at cortical synapses, thus enhancing the susceptibility to absence-like epilepsy (Wang et al., 2015).

#### Pharmacology and Drug Therapy

Common therapeutic strategies adopted to treat different epileptic syndromes include the positive modulation of inhibitory GABAergic transmission, the decrease of excitatory transmission through the inhibition of voltage-gated sodium and calcium channels and other various mechanisms. Regardless of the genetic origin, different AEDs such as benzodiazepines, stiripentol, topiramate, retigabine, and Nav blockers are available on the market and are effective in suppressing the abnormal neuronal firing underlying epileptic seizures (Catterall, 2014; **Table 1**). Despite the numerous available treatments, a large proportion of patients still present drug-resistant seizures and experience frequent adverse drug reactions. Thus new more effective therapeutic options are evaluated by several research groups and pharmaceutical companies to fulfill the need of a personalized medicine, assisted by advances in understanding disease mechanisms and ion channel structures (Catterall, 2014; Walker et al., 2015).

**Table 1:** Central nervous system channelopathies.

Disease	*Gene* (protein)	Pharmacotherapy	Pharmacological perspectives
Epileptic syndromes including GEFS+, SMEI, BFNIS, BFNC, MPSI, ADNFLE, MLC, absence epilepsy and other epileptic encephalopathies (see text for abbreviations)	*SCN1A* (Nav1.1) *SCN2A* (Nav1.2) *SCN3A* (Nav1.3) *SCN8A* (Nav1.6) *SCN1B* (Nav2.1) *KCNA1* (Kv1.1) *KCNA2* (Kv1.2) *KCNC3* (Kv3.3) *KCND2* (Kv4.2) *KCND3* (Kv4.3) *KCNQ2* (Kv7.2) *KCNQ3* (Kv7.3) *KCNH5* (Kv10.1) *KCNJ10* (Kir4.1) *KCNJ11* (Kir6.2) *ABCC8* (SUR1) *KCNMA1* (KCa1.1) *KCNT1* (KCa4.1) *CACNA1C* (Cav1.2) *CACNA1A* (Cav2.1) *CACNA1H* (Cav3.2) *CLCN2* (ClC-2) *GLIALCAM*	Antiepileptic drugs aim at reducing neuronal hyperexcitability with different mechanisms: – Enhancement of GABAergic transmission: benzodiazepines, phenobarbital, valproate, stiripentol, topiramate – Inhibition of glutamatergic transmission: topiramate – Inhibition of Nav channels: phenytoin, carbamazepine, lamotrigine, valproate, topiramate, lacosamide, eslicarbazepine – Opening of K channels: retigabine, acetazolamide – Inhibition of T-type Cav channels: ethosuximide – Inhibition of the synaptic vescicle 2A: levetiracetam – Inhibition of presynaptic Cav2 channels α2δ auxiliary subunit: gabapentin, pregabalin – Inhibition of carbonic anhydrase: topiramate, acetazolamide	– Development of subtype-selective Nav channels ligands with improved safety and efficacy – Development of pharmacological chaperones for folding defective mutants of Nav, Kv and ClC channels – Development of more selective and safer Kv openers

Episodic and spinocerebellar ataxias	*KCNA1* (Kv1.1) *CACNA1A* (Cav2.1) *KCNC3* (Kv3.3) *KCND3* (Kv4.3)	Symptomatic drugs aim at restoring cerebellar functioning and reducing frequency, duration and severity of attacks: – Inhibition of carbonic anhydrase: acetazolamide, in EA1 and EA2 – Inhibition of Kv channels: 4-aminopyridine, first choice in EA2 – Riluzole: Nav channels blocker and SK and TWIK channels opener, in cerebellar ataxias	– Opening of SK channels to restore Purkinje cells pacemaking: chlorzoxazone and 1-EBIO, in EA2 animal models – Kv1.1 dysinactivators

Familial hemiplegic migraine	*CACNA1A* (Cav2.1) *SCN1A* (Nav1.1)	Symptomatic and prophylactic drugs aim at reducing frequency and painful attacks: – Tricyclic antidepressants, β-blockers, triptans, Cav blockers, AEDs, acetazolamide	– Botulinum toxin



Phenytoin, carbamazepine, lamotrigine, and valproate are well-known state- and frequency-dependent non-selective Nav inhibitors, binding to the local anesthetic receptor site in the channel pore, according to the modulated receptor hypothesis, and preferentially stabilizing channels in the non-conducting inactivated state (Catterall and Swanson, 2015). Although they lack Nav isoform specificity, these blockers should allow a selective inhibition of action potential generation in the depolarized and rapidly firing cells that are responsible for epilepsy, leaving unaffected normally functioning tissues. These drugs are widely used for partial and generalized epilepsy but not for absence epilepsy (Catterall, 2014). Among sodium channel blockers recently approved as AEDs, it is worth mentioning lacosamide and eslicarbazepine, that shift the slow inactivation curve of the Nav channel to more hyperpolarized potentials and enhance the maximal fraction of channels that are in the slow inactivated state (Rogawski et al., 2015; Soares-da-Silva et al., 2015). In addition, current efforts in sodium channel pharmacology are focused to develop subtype-selective Nav channel ligands with improved safety and efficacy compared to first generation of Nav blockers. Interestingly, several antidepressant drugs have been shown to block sodium channels, and this action may contribute to their anticonvulsant effects (Igelström and Heyward, 2012). Of note, blockade of voltage-gated sodium channels also results in inhibition of glutamate release, which is associated with anticonvulsant effect.

In Dravet syndrome, as well as in other *SCN1A*-related seizure disorders, sodium channel blockers such as carbamazepine, lamotrigine, phenytoin are contraindicated as they may increase frequency and severity of seizures (Catterall, 2014; Miller and Sotero de Menezes, 2014; Schoonjans et al., 2015). In children with *SCN1A*-related epilepsies, seizure control is particularly critical as they are at high risk for sudden unexplained death in epilepsy. In addition, there are clinical evidences that controlling seizures will reduce the subsequent brain injury and frequent comorbidity with cognitive impairment. In agreement with reduced GABAergic tone in *SCN1A*-related seizures, enhancement of GABAergic transmission in inhibitory interneurons would be the most beneficial therapeutic intervention. Indeed, treatment of conditional *Scn1a^+/-^* mouse model of SMEI with low-dose clonazepam protected against myoclonic and generalized tonic-clonic seizures and completely rescued the abnormal social behaviors and deficits in fear memory (Bender et al., 2012; Oakley et al., 2013). At present, the standard treatment to treat SMEI children in Europe is either the combination of stiripentol, valproate and clobazam, or valproate in combination with topiramate (Miller and Sotero de Menezes, 2014; Schoonjans et al., 2015). Stiripentol has obtained orphan drug status for the treatment of SMEI in Europe (Prunetti and Perucca, 2011). Evidences from an animal model with haploinsufficiency for *Scn1a* and *Scn8a* suggested that reduction in the activity of Nav1.6 channels expressed in excitatory neurons might be an alternative useful option for Nav1.1-associated seizures in SMEI (Chen et al., 2008; de Lera Ruiz and Kraus, 2015). Recently, levetiracetam, which binds to the synaptic vesicle protein SV2A, was shown to be effective in GEFS+, as add-on therapy in SMEI, and in a broad range of epilepsy syndromes (Catterall, 2014).

Even though a selective channel opener might be a particularly effective AED for Nav1-associated syndromes due to loss-of-channel function, no such drug has been developed to date (de Lera Ruiz and Kraus, 2015). Recently, different rescuing approaches have been explored to increase surface expression and restore the correct functioning of sodium channel mutants associated to GEFS+ and SMEI that present folding defects *in vitro* (Bechi et al., 2015). In addition, although sodium channel β subunit-specific drugs have not yet been developed, this protein family is an emerging therapeutic target (O’Malley and Isom, 2015).

Quinidine, an antiarrhythmic and antimalarial drug, proved effective in reversing the increased current of *KCNT1* mutants *in vitro* and in remitting seizures in three patients with *KCNT1* mutations resistant to multiple antiepileptic agents (Lim et al., 2016). However, this drug acts on various cardiac potassium channels and could possibly lengthen the QT interval and lead to cardiac arrest. Thus, development of specific *KCNT1* channel inhibitors with good blood–brain barrier penetration may provide a safer and more effective alternative in affected patients (Lim et al., 2016).

Another strategy to balance excitation and inhibition in the brain consists in incrementing potassium currents, such as KCNQ/K_v_7 currents. The first agent proven to enhance M-current activity was retigabine that has recently been approved as a first-in-class AED for the treatment of resistant partial-onset seizures (Humphries and Dart, 2015). Retigabine stabilizes the K_v_7 open channel conformation and induces a hyperpolarizing shift of the activation curve (Grunnet et al., 2014; Miceli et al., 2015a). Yet, the drug shows limited selectivity among all the neuronal K_v_7 channel subtypes and possesses significant activation of GABA_A_ receptors. Because of short half-life, retigabine requires a high and frequent dosing regimen that, together with the relatively poor selectivity, contributes to relevant adverse effects, including sedation, blue skin discoloration and eye abnormalities. Thus, more selective K_v_7.2 activators are under development (Grunnet et al., 2014).

Among AEDs, ethosuximide is a fist-line treatment of childhood absence seizures (CAE), acting through inhibition of low-threshold T-type calcium channels (comprising Ca_v_3.1, 3.2, and 3.3 channels) that are generally believed to contribute to increasing seizures susceptibility. Perplexingly, about 50% of CAE patients did not respond to ethosuximide, and the majority of the non-responders carry Ca_v_3.2 gain-of-function mutations (Chen et al., 2003; Glauser et al., 2013). Recently, studies from the first animal model of Ca_v_3.2-linked absence epilepsy raised the possibility to reduce slow wave discharges through NMDA and AMPA receptors antagonists but not T-type calcium blockers (Wang et al., 2015).

The gabapentinoid drugs, pregabalin and gabapentin, have emerged in recent years as promising therapeutic options in epilepsy and chronic pain. They target primarily the α2δ auxiliary subunit of the presynaptic Ca_v_2 channels, thereby altering their membrane trafficking and inhibiting excitatory neurotransmission (Catterall and Swanson, 2015).

Current treatment for megalencephalic leukoencephalopathy is only symptomatic, including management of seizures and spasticity with topiramate and carbamazepine (Batla et al., 2011; van der Knaap and Scheper, 2011).

### Cerebellar Ataxias and Migraine

The episodic ataxias are a group of rare autosomal dominant diseases characterized by recurrent attacks of vertigo and cerebellar ataxia (Jen et al., 2007). Episodic ataxia type 1 (EA1) is caused by loss-of-function mutations in the voltage-dependent potassium channel gene *KCNA1* (D’Adamo et al., 2015b). Affected patients display constant myokymia and short episodes of spastic contractions of the skeletal muscles of the head, arms and legs, with loss of both motor coordination and balance. The spectrum of clinical manifestations of the disease also includes cognitive impairments, seizures, blurred vision, neuromyotonia, migraine, short sleep phenotype (Imbrici et al., 2011; D’Adamo et al., 2015b,a). The functional characterization of several K_v_1.1 mutations and the generation of a knock-in animal model of EA1 (Herson et al., 2003), both suggest that increased neuronal excitability and altered GABAergic inputs to cerebellar nuclei likely generate the disease. Episodic Ataxia type 2 (EA2) is the most frequent subtype of episodic ataxia caused by loss-of- function mutations in the *CACNA1A* gene encoding the alpha1A subunit of P/Q type voltage-gated Ca^2+^ channels (Ca_v_2.1) mainly expressed in Purkinje and granule cells in the cerebellum (Jen et al., 2007). EA2 is characterized by longer attacks of ataxia compared to EA1, and is associated with headache and nystagmus both during and between the episodes attacks, but not myokymia (Jen et al., 2007; Graves et al., 2008). The disorder frequently progresses toward a permanent ataxia with cerebellar atrophy. It is generally assumed that ataxia originates from impaired cerebellar neurotransmission (Pietrobon, 2010); emerging data also point to a loss in the precision of cerebellar Purkinje cells (PCs) pacemaking (Walter et al., 2006).

Spinocerebellar ataxias (SCAs) are a very heterogeneous group of autosomal dominant neurological disorders caused by degeneration of the cerebellum and spinal cord. SCAs present a wide range of phenotypes, including cerebellar ataxia, dysarthria, extrapyramidal symptoms, oculomotor disturbance, cognitive impairment, and epilepsy. SCA6 (prevalence: <1/1000,000) is characterized by late onset, slowly progressive cerebellar ataxia (Pietrobon, 2010). It is due to small expansions of a polyglutamine stretch in the C-terminal tail of the Ca_v_2.1 calcium channel, which likely causes an accumulation of Ca_v_2.1 channels in the cytoplasm (Pietrobon, 2010). SCA13 (prevalence: <1/1000,000) is caused by point mutations in the *KCNC3* gene (encoding K_v_3.3 channels), which affect channel function by gain- and loss-of-function mechanisms (Minassian et al., 2012). This channel is quite exclusively expressed in fast spiking neurons, where its dysfunction affects firing frequency and neurotransmitter release. Loss-of-function mutations in the *KCND3* gene (K_v_4.3) have been found in patients with SCA19 and SCA22 (prevalence: <1/1000,000; Duarri et al., 2012; Lee et al., 2012).

Familial hemiplegic migraine type 1 (FHM1) is a rare and severe form of migraine with hemiplegic aura associated with cerebellar deficit in about 50% of patients (Pietrobon and Moskowitz, 2013). FHM1 is due to gain-of-function missense mutations, mostly affecting the pore and the voltage sensor module of the Ca_v_2.1 channel (Pietrobon and Moskowitz, 2013; Jen, 2015). Electrophysiological recordings from cerebellar granule cell of a mouse model of FHM1, bearing the voltage sensor R192Q mutation, showed increased Ca_v_2.1 density and glutamate release and increased susceptibility to cortical spreading depression as the pathophysiologic mechanism underlying migraine with aura (Tottene et al., 2009; Pietrobon, 2010). FHM3 is associated with gain-of-function heterozygous mutations in the *SCN1A* gene likely leading to hyperexcitability of GABAergic interneurons (Dichgans et al., 2005; Cestèle et al., 2013).

#### Pharmacology and Drug Therapy

No specific treatment exists for individuals affected by episodic ataxias type 1 and 2, as openers of neuronal K_v_1.1 and P/Q type calcium channels are dramatically lacking (**Table 1**). Both EA1 and EA2 patients are often treated with acetazolamide, a carbonic-anhydrase (CA) inhibitor, although with variable effectiveness and notable side effects during chronic treatment (such as nephrocalcinosis, hyperhidrosis, paresthesia, muscle stiffening with easy fatigability, and gastrointestinal disturbances; D’Adamo et al., 2015b, Jen, 2015). The mechanisms of action of this drug are still unclear and may include activation of skeletal muscle Ca^2+^-activated-K^+^ (BK) channels or ClC-1 channels (see below Eguchi et al., 2006; Tricarico et al., 2006). Today, 4-aminopyridine is the treatment of choice for EA2. The drug, at the concentrations used, likely targets the K_v_1 channels, possibly the K_v_1.5 subtype, whereas at higher concentrations it blocks a large array of K^+^ channels and is a proconvulsant. Its efficacy in relieving ataxic gaits with only minor side effects was assessed first in a pilot study for EA2 in humans and confirmed in a prospective randomized, double blind, placebo-controlled crossover study in familial EA with nystagmus. Actually, two randomized controlled trials on EA2 with 4-aminopyridine versus acetazolamide are ongoing (EAT-2-TREAT; Strupp et al., 2015).

An alternative approach for the treatment of EA2 may consist in the activation of the widely- distributed, small-conductance Ca^2+^-activated-K^+^ (SK) channels to improve PCs firing. Treatment with 1-ethyl-2-benzimidazolinone or chlorzoxazone, two SK channel activators, efficaciously restored the correct pacemaking activity of PCs and reduced ataxia and dyskinesia in tottering and ducky EA2 mouse models (Walter et al., 2006; Alvina and Khodakhah, 2010a). Actually, also 4-AP was shown to restore PC pacemaking and neurotransmitter release in mouse models of EA2 (Alvina and Khodakhah, 2010b).

Compounds that disrupt Kv1.1 N-type inactivation induced by β1 subunits have been developed and might be useful for reducing neuronal hyperexcitability in diseases, such as epilepsy and neuropathic pain, but could also be adjuvant in diseases presenting with cognitive symptoms of hippocampal origin (Humphries and Dart, 2015).

No specific treatments have been demonstrated effective in progressive SCA (**Table 1**). Acetazolamide is used to alleviate episodes of ataxia, and gabapentin may be beneficial to SCA6 patients (Nakamura et al., 2009; Ilg et al., 2014). Repurposing of existing drugs is an approach that is being increasingly explored. A 1-year randomized, double-blind, placebo-controlled trial with riluzole, the only drug licensed for the treatment of amyotrophic lateral sclerosis, in patients with hereditary cerebellar ataxia of different aetiologies suggests the potential efficacy of this drug (Romano et al., 2015). Riluzole blocks sodium channels, opens SK channels, enhances activity of TWIK-related potassium channel-1 (TREK-1), and reduces glutamate release, thereby decreasing neuronal hyperexcitability. Longer confirmatory studies on larger and disease-specific populations are, however, needed.

The available therapy for all FHM types during an episode of hemiplegic migraine is only symptomatic (**Table 1**). Standard prophylactic drugs for frequent migraine include tricyclic antidepressants, β-blockers, calcium channel blockers, triptans, AEDs, and acetazolamide, but there is no evidence of clinical efficacy on a large scale for any of these medications (Omata et al., 2011; Diener et al., 2015; Jen, 2015).

## Peripheral Nerve Channelopathies

### Pain Syndromes

As in the central nervous system, ion channels play a pivotal role in excitability and communication in the peripheral nervous system (PNS), consisting in the somatic, autonomic, and sensory nervous systems. Genetic ion channelopathies of the PNS encompass gene mutations in the voltage-gated sodium, potassium and transient receptor potential channels (TRP; Miceli et al., 2012; Bennett and Woods, 2014). Many sodium channel subtypes are expressed in the PNS. The Na_v_1.3 channel is expressed mainly during embryogenesis, but *de novo* expression has been reported after nerve injury and inflammation, suggesting a possible role in neuropathic pain. The Na_v_1.1, 1.2, and 1.6 are expressed in both the PNS and CNS, and mutations in these channels result in epileptic diseases (see above), while little is known about possible peripheral symptoms. In contrast, *SCNA9* (Na_v_1.7), *SCNA10* (Na_v_1.8), and *SCNA11* (Na_v_1.9) channels are considered as specific PNS channels (Catterall, 2012). Mutations in these channels cause a series of familial or sporadic pain disorders (Brouwer et al., 2014; Waxman et al., 2014; Habib et al., 2015). Loss of function of Nav1.7 is linked to recessive congenital pain insensitivity or hereditary sensory and autonomic neuropathy (Cox et al., 2006; Yuan et al., 2013). Conversely, gain of function of Nav1.7 leads to a triad of autosomal dominant familial pain syndromes, including inherited erythromelalgia (IEM), paroxysmal extreme pain disorder (PEPD; prevalence <1/1000,000), and painful small-fiber neuropathy (SFN; Yang et al., 2004; Fertleman et al., 2006; Faber et al., 2012a). Functional studies of Na_v_1.7 mutants suggest that IEM mutations essentially enhance channel activation and the channel ramp response, whereas PEPD mutations rather impair fast and/or slow inactivation and produce resurgent currents (Lampert et al., 2014). The SFN mutations induce functional alterations overlapping those of IEM and PEPD. These biophysical defects invariably enhance neuron excitability, which accounts for pain exacerbation. Interestingly, a study recently described a possible link between Na_v_1.7 mutation and the neurodegeneration observed in SFN, because of reverse Na/Ca exchanger-induced Ca^2+^ toxicity (Estacion et al., 2015). Gain of function mutations of the Na_v_1.8 channel also lead to painful SFN, showing faster recovery from inactivation, hyperpolarized activation, or impaired fast inactivation (Faber et al., 2012b; Han et al., 2014). While Na_v_1.7 and Nav1.8 channels allow the rapid upstroke of action potentials in nociceptors, Na_v_1.9 channels have much slower kinetics and are active near the resting potential, thereby lowering the excitability threshold. Gain of function mutations of Na_v_1.9 were recently reported to cause familial episodic pain (Zhang et al., 2013; Huang et al., 2014; Leipold et al., 2015). Quite surprisingly, another Na_v_1.9 mutation was associated to loss of pain sensation, likely due to the concomitant alteration of channel activation and inactivation resulting in a larger window current that, in turn, causes sustained depolarization and inactivation of nociceptors (Leipold et al., 2013, 2015).

Peripheral nerve hyperexcitability seems also related to mutations in potassium channels underlying the M-current, such as R207W/Q in K_v_7.2 (Wuttke et al., 2007). In some cases the disease is attributable to the generation of gating pore currents (Miceli et al., 2012).

Only one mutation has been found in TRPA1 channel to cause familial episodic pain syndrome type I (Kremeyer et al., 2010). The enhanced activation of mutant channel by endogenous mediators likely accounts for the intense pain experienced by carriers.

In addition, besides the pathogenic mutations, some single nucleotide polymorphisms found in various ion channels, including Na_v_1.7, have been shown to contribute to altered pain sensations in various conditions (Bennett and Woods, 2014; Brouwer et al., 2014).

#### Pharmacology and Drug Therapy

The familial pain disorders can be severely disabling and require the use of analgesics in addition to cold compress. At the moment, therapeutic management of these channelopathies is generally very difficult, often requiring combined medication (**Table 2**). Controlled trials of drug effectiveness are lacking, and the choice of drug relies mainly on physician experience. Acetaminophen and non-steroidal anti-inflammatory drugs may give some pain relief, but a significant number of patients get no response (Estacion et al., 2015; Leipold et al., 2015). In addition, the response to neuropathic pain medications, such as antidepressants and gabapentinoids, is quite variable between individuals. Recently, intrathecal ziconotide, a calcium channel blocker, have proved beneficial in a woman with primary erythromelalgia, with reduction of burning pain and net improvement of lower extremity edema (Russo et al., 2015). Targeting Na_v_ channels to modulate chronic/neurpathic pain is a widely used strategy. For example, topical lidocaine patch is considered a first or second-line drug to treat localized neuropathic pain, such as post-herpetic nevralgia or diabetic neuropathy (Finnerup et al., 2015). Also the analgesic effect of the local anesthetic butamben, used through epidural administration to alleviate chronic pain, might be due to modulation of Na_v_ channels (Shulman et al., 1998, 2000; McCarthy et al., 2002; Thériault et al., 2014). The use of sodium channel blockers may appear straightforward to reduce nociceptor hyperexcitability due to gain of function Na_v_ mutations. A study reported a satisfactory response to lidocaine patch in about one-half of 34 patients suffering from primary erythromelalgia (Davis and Sandroni, 2005). A Na_v_1.7 mutation was shown to affect the binding of the local anesthetic, which may explain in part the significant proportion of non-responders (Sheets et al., 2007). Only one IEM mutation has been shown to increase Na_v_1.7 channel sensitivity to mexiletine, likely contributing to the favorable response to the drug observed in this specific patient (Choi et al., 2009). Carbamazepine, a sodium channel blocker indicated for trigeminal neuralgia, was reported to be effective in 14 PEPD patients although the response was incomplete (Fertleman et al., 2007). The drug was also effective in an IEM family members carrying a Na_v_1.7 mutation with enhanced carbamazepine sensitivity (Fischer et al., 2009). One may expect that such studies will help to design a pharmacogenetics strategy to identify the most effective drug for each mutation, as predicted by in silico studies (Yang et al., 2012). Nevertheless, treatment of the PNS channelopathies remains in most cases an unmet medical need.

**Table 2:** Peripheral nervous system channelopathies.

Disease	*Gene* (protein)	Pharmacotherapy	Pharmacological perspectives
**Painful syndromes** Inherited erythromelalgia (IEM), paroxysmal extreme pain disorder (PEPD), painful small-fiber neuropathy (SFN), familial episodic pain syndrome (FEPS)	*SCN9A* (Nav1.7) *SCN10A* (Nav1.8) *SCN11A* (Nav1.9) *TRPA1* (Trpa1)	Symptomatic therapy aims at alleviating pain with different mechanisms of action: – Tricyclic and SNRI Antidepressants – Gabapentinoids – Ziconotide, a calcium channel blocker – Nav blockers such as lidocaine and carbamazepine – Acetaminophen and NSAIDS – Opioids	– Development of potent and/or selective sodium channel blockers for neuropathic and inflammatory pain – Capsaicin or lidocaine patches – Botulinum toxin – Cannabinoids

**Painless syndromes** Congenital insensitivity to pain, Idiopathic small fiber neuropathy	*SCN9A* (Nav1.7)	No drug available	


By demonstrating unambiguously the pivotal role Na_v_1.7, 1.8 and 1.9 channels in pain sensation, the discovery of PNS Na_v_ channelopathies have paved the way for the development of potent and/or selective sodium channel blockers for application in neuropathic and inflammatory pain (Ghelardini et al., 2010; Theile and Cummins, 2011; Bagal et al., 2014; Carbonara et al., 2015). Some of these drugs have entered clinical trials, but whether they will be helpful to treat channelopathies remains to be verified.

## Skeletal Muscle Channelopathies

### Non-dystrophic Myotonias

Non-dystrophic myotonias include myotonia congenita (MC; prevalence 1-9/100,000), paramyotonia congenita (PMC) and SCM. They all share a common symptom, myotonia, that is characterized by impaired muscle relaxation after contraction and is evident as typical “myotonic runs” at EMG (Cannon, 2015).

Myotonia congenita is caused by loss-of-function mutations in the *CLCN1* gene coding for the skeletal muscle chloride channel ClC-1 (Imbrici et al., 2015b). It can be inherited in a dominant (Thomsen) and recessive (Becker) form, this latter presenting with a more severe myotonic phenotype, frequent transient weakness, and sometimes myopathic signs. Myotonia improves with exercise, according to the so-called “warm-up” phenomenon. Alterations of ClC-1 have been reported also in myotonic dystrophies and in Duchenne muscular dystrophy (De Luca et al., 2003; Cardani et al., 2012); although the underlying mechanisms are different, these alterations can account for common membrane hyperexcitability signs. The ClC-1 channel normally dampens membrane excitability and stabilizes the resting membrane potential after an action potential (Jentsch, 2015). Thus, the reduced chloride conductance resulting from MC mutations predispose the sarcolemma to spontaneous action potential runs or abnormal afterdischarges that hamper muscle relaxation after contraction, causing myotonia.

Dominantly inherited missense mutations in the *SCN4A* gene encoding the skeletal muscle voltage-gated sodium channel Na_v_1.4 cause either PMC or SCM (Cannon, 2015). PMC is characterized by early onset muscle stiffness that worsens with cold and exercise (hence paradoxical). Patients often experience muscle weakness that can last several hours. In contrast, SCM presents with different degrees of pure myotonia without weakness. Laryngospasm is a life-threatening symptom reported in neonates carrying some SCM mutations, leading to the description of a new clinical entity called severe neonatal episodic laryngospam (SNEL; Lion-Francois et al., 2010). In heterologous expression systems, sodium channel mutants usually show impaired fast inactivation with enhanced activation or not (Cannon, 2015). Slowed inactivation has been suggested to induce resurgent Na currents due to re-opening of Na^+^ channels during repolarisation (Jarecki et al., 2010). The net effect of these gain-of-function disturbances is an increase in Na^+^ entry into the muscle fibers, which allows repetitive discharges that persist beyond the duration of the stimulus.

#### Pharmacology and Drug Therapy

**FIGURE 2:**
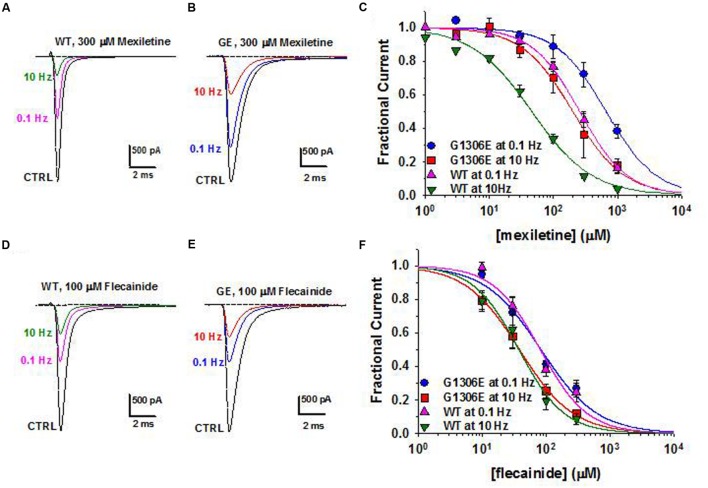
**Pharmacogenetics of myotonic sodium channel mutants. (A,B)** Representative sodium current traces from hNav1.4 WT channels and myotonic G1306E mutants recorded in transfected cells at steady-state before and during application of mexiletine at 0.1 and 10 Hz stimulation frequencies. **(C)** Concentration-effect relationships for mexiletine on WT and G1306E, fitted to a first-order binding function. **(D,E)** Effects of flecainide on sodium currents in the same conditions as in **(A,B)**. **(F)** Concentration-effect relationships for flecainide on WT and G1306E. The G1306E mutation impairs mexiletine inhibition, while leaving flecainide affinity unchanged. Consequently, patients carrying G1306E, who suffer form a severe form of myotonia, obtained significant improvement by shifting treatment from mexiletine to flecainide.

Today, the sodium channel blocker mexiletine represents the first line therapy for the myotonic syndromes, irrespective of the culprit gene (**Table 3**). This drug can be effective in reducing stiffness and transient weakness in non-dystrophic myotonias and myotonic dystrophy (Statland et al., 2012; Lo Monaco et al., 2015). Nevertheless its use is limited by country availability, side effects and limited or null response in some patients (Desaphy et al., 2013b; Matthews and Hanna, 2014; Suetterlin et al., 2015). *In vitro* and *in vivo* studies have shown that the variable clinical efficacy of mexiletine in SCM patients may rely on the different sensitivity of specific mutant channels to the drug (**Figure 2**; Desaphy et al., 2001, 2004, 2013b). Thus, the functional characterization of biophysical defect caused by each mutation could help to predict patients’ responsiveness to a drug and to address the more appropriate therapy. Recently, ranolazine and lacosamide, drugs used to treat angina and epilepsy respectively, have been shown to enhance slow inactivation of sodium channels and thus suggested as an alternative to mexiletine in MC non-responders (Novak et al., 2015). In parallel, other sodium channel blockers on the market for different therapeutic indications, such as riluzole, are under investigation as possible alternatives to mexiletine (Desaphy et al., 2013a, 2014). Finally, a series of structure-activity studies of mexiletine and tocainide derivatives have been performed to develop Nav1.4 channels blockers a 100 times more potent and use-dependent than parental compounds, which may prove useful in myotonic conditions (Talon et al., 2001; De Luca et al., 2004; Carocci et al., 2010; De Bellis et al., 2013; Muraglia et al., 2014).

**Table 3:** Skeletal muscle channelopathies.

Disease	Gene (protein)	Pharmacotherapy	Pharmacological perspectives
Non-dystrophic myotonias including myotonia congenita (MC), paramyotonia congenita (PMC), and sodium channel myotonia (SCM)	*CLCN1* (ClC-1) *SCN4A* (Nav1.4)	Symptomatic (MC) and targeted (PMC and SCM) therapy aims at reducing skeletal muscle hyperexcitability: – Mexiletine (first choice), carbamazepine, flecainide, propafenone are use-dependent Nav channels blockers – Charbonic anydrase inhibitors such as acetazolamide likely increase BK channels activity and ClC-1 channels open probability	– Enhancement of Nav slow inactivation with ranolazine and lacosamide – Repurposing of marketed Nav blockers, such as riluzole – Development of more selective and use-dependent Nav blockers – Development of ClC-1 channel activators and pharmacological chaperones for MC – Development of a pharmacogenetics approach

Periodic paralysis (PP) including hyperkalemic periodic paralysis (HyperPP), hypokalemic periodic paralysis types 1 and 2 (HypoPP1 and 2), Andersen-Tawil syndrome, tyreotoxic periodic paralysis	*SCN4A* (Nav1.4) *CACNA1S* (Cav1.1) *KCNJ2* (Kir2.1) *KCNJ6* (Kir2.6)	Symptomatic therapy aims at reducing frequency and severity of paralytic attacks and at restoring serum K^+^ levels: – Carbonic anhydrase inhibitors such as acetazolamide and dichlorphenamide (orphan drug for PP) likely increase BK channels activity and ClC-1 channels open probability – The β2-agonist salbutamol and glucose/insulin activate Na^+^/K^+^-ATPase and restore serum K^+^ levels in HyperPP – Potassium supplements or K^+^ sparing diuretics in HypoPP – Benzothiazide diuretics such as hydrochlorothiazide in HyperPP	– Development of guanidinium derivatives and other *I*gp blockers in HypoPP – Development of KATP openers selective for skeletal muscle channels – Bumetanide, a loop diuretic that inhibits the Na/K/2Cl co-transporter, was useful in a mouse model of HypoPP



The carbonic anhydrase inhibitor acetazolamide has been used with variable success in myotonia (Benstead et al., 1987; Trudell et al., 1987; Markhorst et al., 2014). One possible mechanism accounting for membrane electrical stabilization by acetazolamide consists in the opening of skeletal muscle BK calcium-activated potassium channels (Tricarico et al., 2006). More recently, the drug was proposed to increase ClC-1 open probability, probably through a change in pH_i_ (Eguchi et al., 2006). It is however possible that the significant percentage of non-responders stems from a reduced sensitivity of some mutant channels to the drug (Desaphy et al., 2013c).

A specific pharmacological treatment for MC patients is lacking. The ideal drug to treat ClC-1 channelopathy should be one able to increase chloride currents, but this goal is far from being achieved. The R(+)-isomer of 2-(*p*-chlorophenoxy) propionic acid has been shown to increase the skeletal muscle chloride conductance (De Luca et al., 1992). However, a direct effect on heterologously expressed ClC-1 channels has not been observed (Pusch et al., 2000). Insulin-like growth factor-1 or taurine, as well as PKC inhibitors, such as staurosporine or chelerythrine are also able to enhance chloride conductance (Pierno et al., 1994, 2003; De Luca et al., 1998). Although they lack a direct therapeutic interest, compounds able to block gCl, such as 9-anthracen carboxylic acid, clofibric acid derivatives, and niflumic acid can be useful pharmacological tools to develop animal models of myotonia or to investigate hClC-1structure-function relationship (Liantonio et al., 2007; Desaphy et al., 2014).

### Periodic Paralyses

Opposed to myotonia are the familial periodic paralyses (PP), which are rare (1/100,000) autosomal-dominant disorders characterized by transient loss of muscle excitability leading to attacks of flaccid paralysis with weakness (Cannon, 2015; Suetterlin et al., 2015). The PP are classified as hyperkalemic PP (hyperPP; prevalence: 1-9/1000,000), hypokalemic PP (hypoPP; prevalence: 1-9/100,000), or Andersen-Tawil syndrome (ATS), and are caused by mutations of sodium (Na_v_1.4), calcium (Ca_v_1.1), and several inward-rectifier potassium channels (K_ir_2.1, K_ir_2.6, and K_ir_3.4). HyperPP is associated with abnormally elevated serum K^+^ ions levels up to 6 mEq/L triggered by ingestion of potassium-rich food, rest after strenuous exercise, and cold exposure. The pathomechanism in hyperPP is explained by the gain-of-function mutations of Na_v_1.4. The mutant channels display impaired inactivation, resulting in persistent sodium influx and cell depolarization, which in turn inactivates Nav1.4 channel (Cannon, 2015). The depolarization in hyperPP amplifies K^+^ ions eﬄux from the muscle through K^+^ channels that elevate the serum K^+^ ions concentration contributing to the paralysis (Khogali et al., 2015). In hypoPP, blood potassium concentration decreases below 3.2 mEq/L triggered by rest after strenuous exercise, by a meal rich in carbohydrates, or by exposure to cold. Familial forms of hypoPP are caused by *SCN4A* (20%) and *CACNA1S* (60%) mutations of positively charged residues in the channel voltage sensor that create an aberrant permeation pathway for H^+^ or Na^+^ ions, resulting in depolarizing cation leak currents named gating pore currents (*I*gp; Moreau et al., 2014). The fiber depolarization in the hypoPP is therefore due to an unbalance between the depolarizing *I*gp and repolarising K_ir_ and sarco-KATP channel currents (Tricarico et al., 2008b). Administration of insulin/glucose activates the 3Na^+^/2K^+^-ATPase causing a marked hypokalemia, which reduces gene expression/activity of K_ir_ channels (Tricarico et al., 2003, 2008b). All these factors contribute to the fiber depolarization that inactivates Na_v_1.4 and Ca_v_1.1 channels with paralysis (Tricarico and Camerino, 2011).

Andersen-Tawil syndrome is a multiorgan disease characterized by PP, cardiac arrhythmias, and skeletal malformations (Tawil et al., 1994). Paralysis may occurs with either hyperkalemia or hypokalemia associated with various heart manifestations including long QT syndrome (LQT7, see below). Mutations in *KCNJ2* cause the disease by suppressing K_ir_2.1 currents and/or enhancing inward currents, due to dominant negative effects or haplo-insufficiency (Davies et al., 2005). Some *KCNJ2* mutations decrease PIP2 sensitivity or exaggerate the inhibitory effects of intracellular Mg^2+^ or proton. A novel mutation of *KCNJ5* encoding K_ir_3.4 channel exerts a dominant negative effect on K_ir_2.1 in heart and skeletal muscle of ATS patients (Kokunai et al., 2014).

Thyrotoxic periodic paralysis (TPP), associated to hyperthyroidism, is the most common cause of hypokalemic flaccid muscle paralysis in adults. Mutations of *KCNJ18* encoding the K_ir_2.6 channel have been found in a few cases of TPP and of sporadic PP (SPP) not necessarily associated to thyrotoxicosis (Ryan et al., 2010; Cheng et al., 2011). The mutants show reduced or no current and exert dominant negative inhibition of wild-type K_ir_2.6 and K_ir_2.1 channels. The K_ir_ down-regulation predisposes to hypokalemia-induced paradoxical depolarization during attacks. Other susceptibility genetic variants for TPP and SPP include rs623011 and SUR1Ala1369Ser. The former reduces K_ir_2.1 channel transcription in skeletal muscle independently of thyroid hormone (Jongjaroenprasert et al., 2012), while the latter encodes an auxiliary subunit of KATP channel in pancreatic beta cells, fast twitching muscle fibers and heart (Wheeler et al., 2008; Rolim et al., 2014).

Very recently, the spectrum of muscle channelopathies with pronounced weakness has been widened by the discovery of novel Na_v_1.4 mutations causing congenital myasthenic syndrome with PP or severe fetal hypokinesia (Zaharieva et al., 2015; Habbout et al., 2016).

#### Pharmacology and Drug Therapy

Drug therapy of PP can be separated into two categories, treatments used for acute attacks and prophylactic treatments (**Table 3**). In hyperPP, acute attacks may respond to inhaled salbutamol or glucose/insulin therapy that exert their repolarizing actions by activating the 3Na^+^/2K^+^-ATPase and restoring the serum K^+^ levels. In hypoPP, acute attacks are usually treated with oral potassium chloride glucose-free. The treatment of the hyperkalemia or hypokalemia can be successfully achieved by administrating benzothiazide diuretics (hydrochlorothiazide) or K^+^ sparing diuretics, respectively. The most effective medication for prevention of attacks in both disorders remains the carbonic anhydrase inhibitors, acetazolamide and dichlorphenamide. Dichlorphenamide has been recently designed as orphan drug for the treatment of primary periodic paralyses. A very recent clinical trial demonstrated significant efficiency of dichlorphenamide in hypoPP but not hyperPP (Sansone et al., 2016). These drugs ameliorate paralysis reducing frequency of the attacks and vacuolar myophathy in human and animal models of hypoPP (Tricarico et al., 2006, 2008a; Matthews and Hanna, 2014). One of the main mechanisms responsible for their therapeutic effects relies on their capability to open calcium-activated potassium BK channels in muscle fibers (Tricarico et al., 2006, 2013; Dinardo et al., 2012). Yet, in some hypoPP patients, acetazolamide fails to prevent paralysis and may show detrimental effects. For instance, none of the patients with glycine substitutions, i.e., R528G, R1239G, or R672G, respond satisfactorily to acetazolamide but are still responsive to dichorphenamide (Bendahhou et al., 2001; Sternberg et al., 2001; Kim et al., 2007).

Recently, bumetanide, a loop diuretic that inhibits the Na^+^/K^+^/2Cl^-^ co-transporter, proved effective in preventing weakness attacks in a mouse model of hypoPP, thereby representing a novel symptomatic strategy (Wu et al., 2013).

The treatment of ATS is complicated by the need to address two distinct phenotypes, PP and cardiac arrhythmias (Nguyen et al., 2013). PP therapy includes minimizing known triggers and prophylaxis with acetazolamide. Potassium supplementation may also prevent paralytic attacks while simultaneously shortening the QT interval. Treatment of cardiac arrhythmias forecasts the use of β-blockers or flecainide. No specific treatment is available to address muscle weakness in TPP. Acetazolamide should not be used in these patients due to risk of adrenergic symptoms aggravation. Drugs targeting KATP and/or K_ir_ channels in skeletal muscle may be of interest to treat PP. The available KATP channel openers show a poor tissue selectivity and Kirs openers are not available (Humphries and Dart, 2015). Thus, KATP channel openers have been proposed in PP but their use is limited by the side effects (Tricarico and Camerino, 2011; Tricarico et al., 2012). The search for KATP channel openers acting selectively in skeletal muscle is underway (Rolland et al., 2006; Tricarico et al., 2008a, 2012).

One logical strategy to treat PP may consist in blocking the *I*gp, without affecting normal channel gating, however no drug is currently available. A few gating pore blockers have been tentatively identified, including divalent and trivalent cations such as gadolinium (Sokolov et al., 2007, 2010; Struyk and Cannon, 2007). Some guanidinium derivatives block *I*gp but in millimolar concentration range. Differences in the environment surrounding the gating pores may however affect drug responses to this class of compounds (Moreau et al., 2014). In addition, natural toxins can represent interesting lead compounds as *I*gp blockers, such as those able to stabilize the voltage sensor (Moreau et al., 2014).

## Heart Channelopathies

### Heart Arrhythmias

Cardiac channelopathies encompass a large spectrum of heart diseases characterized by arrhythmic events, conduction defects, and cardiomyopathies (Campuzano et al., 2015; Spears and Gollob, 2015). They are among the leading causes of sudden cardiac death (SCD) in young people, with a marked male prevalence. The four principal groups of proarrhythmic channelopathies are long QT syndrome (LQTS; prevalence: 1/2,500), short QT syndrome (SQTS), Brugada syndrome (BrS; prevalence: 1/3,300 to 1/10,000), and catecholaminergic polymorphic ventricular tachycardia (CPVT; prevalence: 1/10,000). Besides the four main cardiac channelopathies, ion channel gene mutations have been linked to a plethora of rare familial cases of cardiomiopathies. For instance, *SCN5A* mutations can cause not only LQT3 and BrS, but also cardiac conduction disease, sick sinus syndrome, atrial fibrillation, or dilated cardiomyopathy (Remme and Wilde, 2014).

The first heart channelopathy to be discovered was a congenital form of long QT syndrome (LQT1) due to mutations in *KCNQ1* encoding the main subunit (K_v_7.1) of the K channel responsible for the slow delayed rectifier current (I_Ks_) in cardiomyocytes (Wang et al., 1996). LQT1 mutations induce a loss of function of K_v_7.1, which delays action potential termination and increases the electrocardiogram QT interval longer than 470/480 ms (male/female), thereby increasing the risk of early after discharges (EADs), ventricular arrhythmia, torsades de pointes, seizures, syncope, and sudden death. Since then, at least fifteen genetic subtypes of LQTS have been reported, including nine forms linked to mutations in genes encoding ion channel subunits, while the others are caused by mutations in proteins that secondarily alter ion channel function or calcium signaling (Spears and Gollob, 2015). LQTS mutations induce either loss of function of K^+^ channel subunits or gain of function of Na^+^ or Ca^2+^ channels.

Conversely, gain of function of K^+^ channels or loss of function of Ca^2+^ channels accelerates the action potential termination, thereby shortening the QT duration (Brugada et al., 2004; Betzenhauser et al., 2015). SQTS is a rare but very severe condition characterized by a QT interval minor to 350/360 ms, which eventually leads to SCD during rest, sleep, or exercise at an early age. Very recently, carnitine deficiency due to mutations in the *SLC22A5* gene encoding the ubiquitary membrane carnitine transporter OCTN2, have been associated to SQT, likely due to secondarily increased K^+^ channel activity (Roussel et al., 2016).

The BrS is characterized by various ECG alterations, especially an elevation of the ST segment followed by a negative T wave (Havakuk and Viskin, 2016). The more frequent mutations are found in *SCN5A* gene encoding Na_v_1.5 sodium channel (Chen et al., 1998). Mutations in *SCN5A* as well as *SCN1B* or *SCN3B*, both encoding auxiliary β-subunits, all reduced Na^+^ currents. A reduction of I_Na_ is also involved in BrS due to mutations of the glycerol-3-phosphate dehydrogenase *GPD1L* gene. Loss of function mutations are found in genes coding for the L-type Ca^2+^ channel in individuals showing overlapping BrS and SQT. Familial dysfunction of the sinoatrial and atrioventricular nodes has been also associated with mutations in *HCN4* channels, cationic channels involved in pacemaker activity (Milanesi et al., 2015). Loss of function in *HCN4* have been observed in rare BrS cases. One gain-of-function mutation in the same channel, R504Q, has been found in a family with inappropriate sinus tachycardia (IST), a syndrome characterized by abnormally fast sinus rate and multisystem symptoms (Baruscotti et al., 2015). Gain of function of the *KCNE3* β-subunit of K^+^ channels responsible for the transient outward current I_to_ are found in less than 1% of BrS cases.

The CPVT is characterized by ventricular arrhythmias triggered by physical exercise, emotional stress, or catecholamine administration, in the absence of structural heart disease. Without treatment, CPVT has a high mortality rate. Mutations in the RyR2 ryanodine receptor, an intracellular calcium channel, were the first identified to cause CPVT (Priori et al., 2002). Mutations in other proteins involved in Ca^2+^ handling, including calsequestrin-2, triadin, and calmodulin, have been also reported.

It is also worth to note that some of these channelopathies can affect different organs; for instance, LQT7 syndrome caused by *KCNJ2* mutations corresponds to Andersen-Tawil syndrome, which is characterized by a triad of ventricular arrhythmias, periodic paralysis, and dysmorphic features (see above). Similarly, *CACNA1C* mutations cause LQT8 in Timothy syndrome.

#### Pharmacology and Drug Therapy

In cardiac channelopathies, syncope is often the first presenting symptom and the risk of SCD is very high. The genetic testing can give invaluable information to support diagnosis in the patient and prognosis in familiars, and to address treatment in both. The management of syncope survivors and relatives at high risk often consists in the use of an implantable cardioverter defibrillator (ICD) to correct or interrupt life-threatening arrhythmias.

In some cases, such as SQTS and BrS, ICD is the only proven therapy. Only a preliminary study suggests that quinidine may be effective in SQT1, likely due to its ability to block open Herg channels (Gaita et al., 2004). In BrS, either quinidine or isoproterenol, which increases the L-type calcium current, may be used to manage refractory and acute arrhythmias, but data are lacking to recommend these drugs as ICD alternative (Priori et al., 2013). Intravenous class 1 antiarrhythmic drugs, such as flecainide or ajmaline, are used as provocative tools of ST elevation for BrS diagnosis.

In both LQTS and CVPT, emotional stress and intense exercise play a prominent role in triggering ventricular arrhythmias. Thus the main pharmacological approach consists in the use of β-adrenoceptor antagonists to reduce sympathetic or adrenergic stimulation. The preferred drug in CPVT is nadolol, a long acting β-blocker lacking intrinsic sympathomimetic activity (Priori et al., 2013). To obtain a better response, high dosage and strict compliance to therapy are required, together with exercise restriction. Flecainide, an antiarrhythmic with mixed action on Na_v_1.5 and RyR2 channels, may be added to prevent breakthrough arrhythmias on β-blockers. An integrated approach to the diagnosis, risk stratification, and genotype- and phenotype-guided management of patients with LQTS has been recently provided for by Giudicessi and Ackerman (2013). Nadolol and propranolol are recommended for the treatment of all LQTS subtypes. Blockers of late Na current, such as mexiletine or ranolazine, may be used as add-on therapy for LQT3 (Ruan et al., 2007; Moss et al., 2008). However, some SCN5A mutations impair mexiletine effects and the drug may favor Na channel trafficking to the membrane, thus raising concerns about the indiscriminate use of sodium channel blockers in LQT3 without careful individual testing (Ruan et al., 2007, 2010). In case of unsatisfactory response or non-compliance to β-blockers, the left cardiac sympathetic denervation (LCSD) may prove useful in both CPVT and LQTS, although randomized control trials to evaluate the risk/benefit of this treatment are lacking.

No gene mutation have been identified in most cases of SCD, thus it is likely that the number of cardiac channel mutations will continue to increase. Despite the large number of ion channels causing cardiac arrhythmias, the therapeutic options are limited and based mostly on symptomatic treatments, with only rare exceptions (**Table 4**). It is expected that future studies will be dedicated to the search of gene- or mutation-specific approaches (El-Sherif and Boutjdir, 2015).

**Table 4:** Heart channelopathies.

Disease	Gene (protein)	Pharmacotherapy	Pharmacological perspectives
Long QT syndrome (LQTS)	*KCNQ1* (Kv7.1) *KCNH2* (HERG) *KCNE1* (Mink) *KCNE2* (MiRP1) *KCNJ2* (Kir2.1) *KCNJ5* (Kir3.4) *SCN5A* (Nav1.5) *SCN4B* (Nav2.4) *CACNA1C* (Cav1.2) Non-channel: *AKAP9* (yatio) *CALM1* (calmodulin) *CALM2* (calmodulin) *CAV3* (caveolin) *SNTA1* (syntrophin a1) *ANKB* (ankyrin B)	Symptomatic therapy aims at restoring normal heart rhythm: – Implantable cardioverter defibrillator (ICD) – β-adrenoceptor antagonists, such as nadolol and propranolol, plus flecainide. – Mexiletine or ranolazine can be used as add-on therapy in LQT3 – Left cardiac sympathetic denervation (LCSD) when β-blockers fail	Gene- and mutation- specific drugs

Short QT syndrome (SQTS)	*KCNH2* (HERG) *KCNQ1* (Kv7.1) *KCNJ2* (Kir2.1)	– Implantable cardioverter defibrillator (ICD) – Quinidine, a class I antiarrhythmic, in SQT1 owing to HERG channels block	

Brugada syndrome (BrS)	*SCN5A* (Nav1.5) *SCN1B* (Nav2.1) *SCN3B* (Nav2.3) *KCNE3* (MiRP2) *CACNA1C* (Cav1.2) *CACNB2* (Cavβ2) *HCN4* (Hcn4)	Symptomatic therapy aims at restoring normal heart rhythm: – Implantable cardioverter defibrillator (ICD) is the only available option – Quinidine (K channel blocker) or isoproterenol (L-type calcium current activator) are used for acute arrhythmias. Their use as an alternative to ICD remains to be verified	Gene- and mutation-specific therapy

Catecholaminergic polymorphic ventricular tachycardia (CPVT)	*RYR2* (RyR2) Non-channel: *CASQ2* (calsequestrin-2) *CALM1* (calmodulin-1) *TRDN* (triadin)	Symptomatic therapy aims at restoring normal heart rhythm: – high doses of β-blockers, such as nadolol, plus flecainide – Left cardiac sympathetic denervation (LCSD) where β-blockers fail	– Gene- and mutation- specific drugs – Gene therapy in mouse models



## Kidney Channelopathies

### Bartter’s Syndrome

Bartter’s syndromes (BS; prevalence: 1/1000,000) are a heterogeneous group of rare salt loosing tubulopathies confined mainly at the level of thick ascending limb (TAL) of Henle’s loop (Loudon and Fry, 2014). BS type III is due to loss of function mutations of *CLCNKB* gene and is characterized by polyuria, polydipsia, hypokalemia, alkalosis, hypercalciuria and frequently nephrocalcinosis (Simon et al., 1997; Krämer et al., 2008). CLC-Kb chloride channels are expressed, together with their accessory subunit, barttin, at the basolateral membrane of TAL and distal convoluted tubule (DCT), where they permit NaCl reabsorption, and in the stria vascularis of the inner ear, where they contribute to the mechanism for K^+^ secretion into endolymph (Estévez et al., 2001). Deletion, nonsense, and missense *CLCNKB* mutations have been reported, which usually cause a disruption of channel trafficking to the plasma membrane or a reduction of channel open probability (Andrini et al., 2015). Missense and nonsense mutations in *BSDN*, encoding barttin, result in antenatal BS type IV, the most severe BS form, characterized by excessive polyhydramnios and prematurity with early failure to thrive and sensorineural deafness (Janssen et al., 2009; de Pablos et al., 2014). In addition, impaired palmitoylation of some barttin mutants may play a role in chloride channel dysfunction (Steinke et al., 2015). Conversely, gain-of-function polymorphisms in *CLCNK* genes have been reported to be associated with salt retention and hypertension (Barlassina et al., 2007).

The K_ir_1.1 (or ROMK) channel encoded by *KCNJ1* is the major secretory channel in the kidney (Welling and Ho, 2009). ROMK loss-of-function mutations cause BS type II, characterized by antenatal presentation of polyhydramnios, premature delivery, profound postnatal polyuria, hypokalaemia, hyponatraemia and hypercalciuria, as well as secondary nephrocalcinosis later in life (Simon et al., 1996; Brochard et al., 2009). The defective function of ROMK channel mutants impairs K^+^ movement from intracellular compartment to TAL lumen (Boim et al., 1995).

#### Pharmacology and Drug Therapy

Management of patients with BS is oriented toward acute and chronic therapy of presenting complications and abnormalities (Nascimento et al., 2014; **Table 5**). Fluid and electrolyte replacement led to an immediate amelioration of the symptoms. Long-term treatment in BS consists of a high-salt diet, potassium and magnesium replacement, and potassium-sparing diuretics, such as spironolactone/eplerenone and amiloride. In addition, volume depletion leads to upregulation of prostaglandin E2 by both renal and non-renal mechanisms. Indomethacin, a cyclooxygenase inhibitor, has been used extensively in the treatment of BS and proved efficient in increasing height and body weight, and reducing hyperfiltration. It can frequently induce gastrointestinal complications, such as gastritis, bleeding ulcers, and necrotizing enterocolitis, especially in infants. Definitely, BS therapy remains empirical with one limiting factor represented by the drug-induced risk of progressive renal damage, which can lead to chronic renal failure (Unwin and Capasso, 2006). The identification of therapy of targeting the molecular defect is therefore urgently needed.

**Table 5:** Kidney channelopathies.

Disease	Gene (protein)	Pharmacotherapy and mechanisms of action	Pharmacological perspectives
Bartter’s syndrome (BS) including types II–IV	*CLCNKB* (ClC-Kb), *BSDN* (Barttin), *ROMK1* (Kir1.1)	Symptomatic therapy aims at restoring electrolyte balance: – Potassium-sparing diuretics such as spironolactone/eplerenone and amiloride restore serum K^+^ concentrations – Indomethacin, a COX inhibitor, reduces PGE2 production – Potassium and magnesium supplements restore electrolyte balance	– Development of selective channel openers through a pharmacogenetic approach – Development of pharmacological chaperones

Dent disease type 1	*CLCN5* (ClC-5)	Symptomatic therapy aims at restoring electrolyte balance: – Thiazide diuretics ameliorate hypercalciuria – High citrate and fluid intake control hypercalciuria – Vitamin D counteracts rickets in clinical cases with additional bone disease	– Development of CLC-5 activators – Development of pharmacological chaperones

EAST/SESAME syndrome	*KCNJ11* (Kir4.1)	Symptomatic therapy aims at restoring electrolyte balance and remitting seizures: – Antiepileptic drugs – Indometacin – Oral potassium	



**FIGURE 3:**
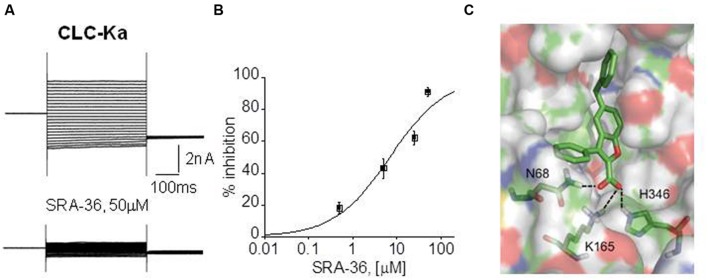
**Electrophysiological drug testing on expressed chloride channels and docking simulation. (A)** Representative CLC-Ka patch-clamp recordings from transfected HEK293 cells, before and after the application of a synthetic compound, SRA-36. **(B)** Dose-response curve of CLC-Ka current inhibition by SRA-36 measured at +60 mV. **(C)** Model of the SRA-36 top-scored docking pose resulting from Induced Fit Docking simulations using a homology model of ClC-Ka modeled on the crystal structure of ClC-ec1.

NFA, a non-steroidal anti-inflammatory drug belonging to fenamate, resulted the unique molecule able to activate CLC-K channel expressed in Xenopus oocytes (Liantonio et al., 2008). Although this effect was not reproduced in mammalian cells (Imbrici et al., 2014), NFA may represent a lead compound to create novel optimized ligands to treat BS types III and IV. Despite the lack of CLC-K openers, CLC-K selective blockers exist and may have a therapeutic potential in hypertension (**Figure 3**). Preclinical studies performed in normotensive and hypertensive rats showed that *in vivo* administration of small-molecules inhibitors of CLC-K channels induced diuretic and antihypertensive effects with no toxic effect (Liantonio et al., 2012, 2016; Imbrici et al., 2014).

Similarly to CLC-K, molecular pharmacology of K_ir_1.1 (ROMK) remains quite limited. No K_ir_ channel opener is known (Humphries and Dart, 2015). Since ROMK is expressed in both TAL and CCD, inhibitors may provide superior diuretic/natriuretic efficacy as compared to loop diuretics, which exert their effects in TAL only. A high throughput screening study identified 1,4-bis(4nitrophenethyl)piperazine derivatives as potent ROMK inhibitors (Garcia and Kaczorowski, 2014). In normotensive animals, short-term oral administration of one of these derivatives caused concentration-dependent diuresis and natriuresis that were comparable to hydrochlorothiazide (Garcia et al., 2014).

### Dent’s Disease

Mutations in *CLCN5* gene cause a subtype of Dent’s disease type 1 (prevalence: 250 reported families), a renal tubular disorders characterized by low molecular weight proteinuria, hypercalciuria, nephrolithiasis, nephrocalcinosis, and progressive renal failure (Lloyd et al., 1997; Hodgin et al., 2008). *CLCN5* encodes the CLC-5 protein, which functions as an electrogenic 2Cl^-^/H^+^ antiporter (Picollo and Pusch, 2005). In human kidney, CLC-5 is expressed in proximal tubule (PT), thick ascending limb, and intercalated cells of collecting ducts. In PT cells, CLC-5 co-distributes with the vacuolar H^+^-ATPase in early endosomes, which are responsible for the reabsorption and processing of albumin and low molecular weight proteins filtered by the glomerulus. Thus, the disease is essentially due to defective receptor-mediated endocytosis causing a generalized dysfunction of PT cells. The two constitutive CLC-5 knock-out mouse models (Piwon et al., 2000; Wang et al., 2000), both exhibit the most common features of Dent’s disease, but differ in other characteristics, potentially reflecting the phenotype variability observed in patients. The use of a knock-in mouse harboring a point mutation that converts the exchanger into an uncoupled Cl^-^ channel discloses the importance of chloride concentration for organelle physiology (Novarino et al., 2010). The recent analysis of a large cohort of patients confirmed that *CLCN5* mutations can be divided into three different functional classes affecting the dimerization process, helix stability or ions transport (Mansour-Hendili et al., 2015; Pusch and Zifarelli, 2015).

#### Pharmacology and Drug Therapy

The current care of patients with Dent’s disease is supportive, focusing on the prevention of nephrolithiasis relying mainly on generous liquid intake (**Table 5**). Thiazide diuretics can be used to treat hypercalciuria, although significant adverse events, including hypovolemia and hypokalemia related to primary tubulopathy have been reported (Raja et al., 2002). Similarly, treatment with vitamin D must be cautious since it may increase hypercalciuria. Studies performed on CLC-5 deficient mice suggest that a high-citrate diet can preserve renal function and slow progression of renal disease (Cebotaru et al., 2005). A major future challenge will be to search for specific molecules able to restore CLC-5 mutant function in Dent’s disease patients, but CLC-5 ligands are lacking. Available ligands for other CLC family members could serve as starting molecular structures to reach such an objective.

#### EAST/SeSAME Syndrome

EAST/SeSAME syndrome (seizures, sensorineural deafness, ataxia, mental retardation, and electrolyte imbalance syndrome; prevalence: <1/1000,000) is a multiorgan disorder that presents with a unique set of symptoms including epilepsy, ataxia, mental retardation, hearing loss, and electrolyte imbalance related to renal salt loss (Bockenhauer et al., 2009). It is caused by missense or deletion mutations in *KCNJ10* (K_ir_4.1 channels) that compromise the function of homomeric K_ir_4.1 expressed in kidney DCT, glial cells in the brain and spinal cord, and in the inner ear (Sala-Rabanal et al., 2010; Cross et al., 2013).

#### Pharmacology and Drug Therapy

No specific therapy exists for affected patients (**Table 5**). Seizure control is achieved with typical AEDs. Potassium supplementation accompanied by indometacin administration has also been reported (Cross et al., 2013).

## Endocrine Channelopathies

### Neonatal Diabetes Mellitus and Congenital Hyperinsulinism

In pancreatic beta cells, the main ATP-sensitive K^+^ channel (KATP) complex is formed by the assembly of Kir6.2/SUR1 subunits (Babenko et al., 2000). This channel regulates insulin secretion in response to glucose challenge, and is involved in genetic diseases associated with insulin/glucose dismetabolism. Loss-of-function mutations in *KCNJ11* and *ABCC8* genes underlie familial congenital hyperinsulinism, while gain-of-function mutations and metabolic uncoupling are associated to neonatal diabetes mellitus (prevalence: 1/300,000-400,000) and DEND syndrome with developmental delay, epilepsy and neonatal diabetes (McTaggart et al., 2010; Arnoux et al., 2011).

#### Pharmacology and Drug Therapy

It is known that SUR1 subunit carries the binding sites for the KATP channel blockers, such as glibenclamide, tolbutamide, glimepiride, repaglinide, and nateglinide, acting as insulin-releasing agents in diabetes therapy, and for the KATP channel opener diazoxide used as a hyperglycaemic drug (Babenko et al., 2000). The most used medications in the treatment of congenital hyperinsulinism are diazoxide and somatostatin analogs (Welters et al., 2015; **Table 6**). While insulin therapy is commonly prescribed in neonatal diabetes, glibenclamide is effective in resolving the CNS and peripheral symptoms in patients carrying *KCNJ11* and *ABCC8* mutations replacing insulin therapy (De Franco et al., 2015). However, patients carrying the Gly334Val *KCNJ11* mutation, are not responsive to this drug (Lau et al., 2015).

**Table 6:** Endocrine channelopathies.

Disease	Gene (protein)	Pharmacotherapy	Pharmacological perspectives
Familial congenital hyperinsulinism	*KCNJ11* (Kir6.2) *ABCC8* (SUR1)	Diazoxide, a hyperglycaemic drug that opens KATP channels, and somatostatin analogs reduce insulin release in congenital hyperinsulinism

Neonatal diabetes mellitus and DEND syndrome	*KCNJ11* (Kir6.2) *ABCC8* (SUR1)	Insulin and glibenclamide, a hypoglycaemic drug that blocks KATP channels, increase insulin levels in neonatal diabetes mellitus

Osteopetrosis	*CLCN7* (ClC-7) *OSTM1* (ClC-7 accessory subunit Ostm1)	Therapy aims at increasing bone resorption: – Hematopoietic stem cell transplantation – Calcitriol and interferon γ – Corticosteroids	Development of selective compounds able to open/activate ClC-7 or to slow the accelerated activation of some ClC-7 mutants

### Osteopetrosis and Other Bone Abnormalities

Osteopetrosis (OP; prevalence: 1/250,000) is a heterogeneous genetic condition characterized by increased skeletal mass and results from reduced function of osteoclasts and impairment of bone resorption. Defects in *TCIRG1*, encoding a vacuolar-ATPase subunit, account for nearly 50% of all patients with OP and cause an impairment of vesicle trafficking and lysosomal acidification in osteoclasts leading to bone resorption defect (Sobacchi et al., 2013). More rarely, dominant or recessive mutations are found in *CLCN7* or *OSTM1* genes, encoding the lysosomal 2Cl^-^/H^+^-transporter CLC-7 or its auxiliary subunit Ostm1 (Kornak et al., 2001; Kasper et al., 2005; Leisle et al., 2011). Most CLC-7 mutations induce a loss of channel function; several dominant mutations were shown to accelerate the voltage gating of CLC-7. In mice, the disruption of CLC-7 mimics severe OP with retinal and CNS degeneration as in the recessive form of human OP (Kornak et al., 2001; Weinert et al., 2014). Skeletal dysplasia and defects in bone ossification are associated with gain of function mutations in the calcium-permeable TRPV4 ion channel gene, which result in an increased basal intracellular Ca^2+^ levels (Nilius and Voets, 2013).

#### Pharmacology and Drug Therapy

As osteoclasts are of hematopoietic origin, hematopoietic stem cell transplantation (HSCT) is the only curative treatment for various forms of recessive osteopetrosis in early life, but this is associated with a high incidence of adverse effects such as graft failure, hepatic and pulmonary toxicity, and veno-occlusive disease (Orchard et al., 2015). Otherwise, OP treatment is mainly supportive (**Table 6**). Calcitriol, by modulating blood calcium level, increases osteoclasts activity and interferon γ increases bone resorption, causing a reduction in trabecular bone area and an increase in bone marrow space (Key et al., 1995). Undergoing research aims at identifying selective compounds able to open/activate CLC-7 or to slow the accelerated activation of CLC-7 observed in several patients, and siRNA against specific CLC-7 mutants can be feasible in some therapeutically neglected form of osteopetrosis (Capulli et al., 2015). Conversely, CLC-7 inhibitors as well as anti-CLC-7 antibodies could be used to reduce bone resorption in osteoporosis (Zhao et al., 2009; Ohgi et al., 2011). Proof of concept of gene therapy efficacy has been obtained in mice, in which neonatal transplantation of genetically modified stem cell, using a lentiviral vector expressing murine *TCIRG1*, led to improved bone resorption (Moscatelli et al., 2013). Encouraging preclinical treatments for OP include in utero stem cell treatment, RANK ligand replacement therapy, and denosumab for post-HSCT hypercalcaemia (Sobacchi et al., 2013). A pharmacological block of TRPV4 has been also proposed as a potential therapeutic strategy (Nilius and Voets, 2013).

## Future Perspective in Drug Discovery

**FIGURE 4:**
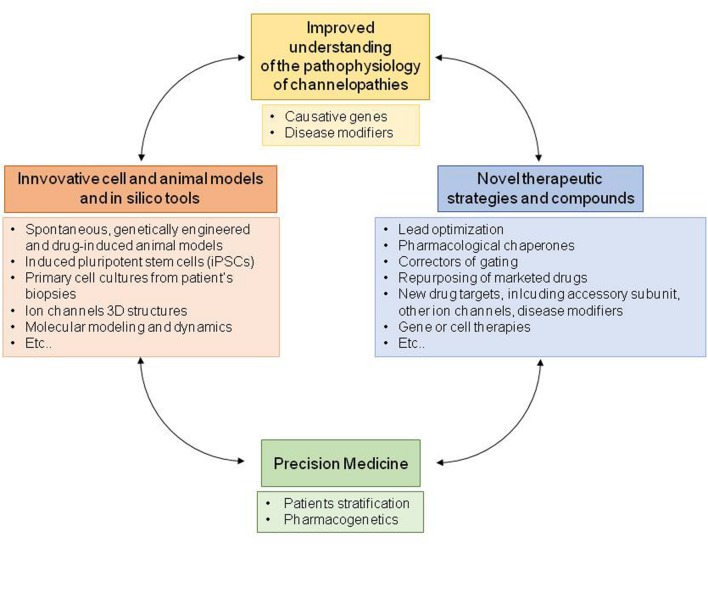
**Schematic diagram illustrating the process of drug discovery, from the analysis of disease pathophysiology to development of a precision medicine**.

The pharmacotherapy of ion channelopathies is a challenging issue in clinical management. To date, only a few channelopathies can benefit from a targeted therapy while, in the majority of cases, the current approaches rely on symptomatic and often empiric medications limited by extensive drug resistance and adverse events (Desaphy et al., 2013b; Lo Monaco et al., 2015). Deciphering the exact pathogenetic mechanism for every channelopathy is fundamental to design therapies tailored upon patients’ requirements. The improvement and generation of disease-related cell and animal models as well as the development of innovative pharmacological strategies greatly help to fulfill this aim. Below, we report some examples of the most remarkable attempts in this direction (**Figure 4**).

### Disease-related Models

Animal models that recapitulate human channelopathies have been pivotal for understanding the underlying pathophysiology and for subsequent preclinical studies (De Luca, 2012; Desaphy et al., 2014; Fratta and Hanna, 2015; Rubinstein et al., 2015; Zou, 2015). When possible, human cell-based approaches, such as primary cell culture from patients’ biopsies and induced pluripotent stem cells (iPSCs), may represent another fascinating opportunity to understand human diseases, properly identify disease pathways and conduct high-throughput drug screening. Patients’ muscle biopsies have been recently employed to investigate the mechanisms of the variable expressivity of myotonia congenita phenotypes (Portaro et al., 2015). In a gene expression study on myotonic patients’ biopsies, the greater expression of the Na^+^ channel β1 subunit and the total lack of potassium channel subunit MiRP2 were correlated with the clinical severity of the Becker myotonic phenotype of the affected individuals (Portaro et al., 2015). This study not only provides evidence for a role of *KCNE3* and *SCN1B* genes as interesting predictors of myotonic phenotype, but also suggests new targets for drug development. As the availability of patients’ biopsies is often poor, the possibility to reprogram somatic cells derived from patients into pluripotent stem cells that can be differentiated *in vitro* is emerging as an appealing opportunity for modeling channelopathies. Recently, human skin cells from Timothy syndrome (TS) patients were reprogrammed into iPSCs and subsequently differentiated into cardiac muscle cells or neurons (Krey et al., 2013; Yazawa and Dolmetsch, 2013; Paşca et al., 2014). Results from these studies showed that TS ventricular-like cardiomyocytes exhibited deficits in contraction, electrical signaling and calcium handling, and that the cyclin-dependent kinase inhibitor and atypical Cav channel blocker roscovitine could successfully rescue this cellular phenotype. Similarly, roscovitine proved efficacious in restoring the neuronal phenotype in cortical neurons derived from TS patients using the same approach (Krey et al., 2013). *In vitro* iPSCs-based models of cerebellar ataxia and Andersen-Tawil syndrome have also been established to recapitulate the complex *in vivo* events underlying these dysfunctions (Watson et al., 2015; Pini et al., 2016). Although fascinating, iPSCs technology is still challenging, one of the most crucial issue being the need to establish a reliable disease phenotype that can be reproduced in quite large quantities *in vitro*. However, once established, it could provide an invaluable resource for drug target identification and drug screening (Skalova et al., 2015; Watson et al., 2015).

### Pharmacological Chaperones

One promising approach to treat protein-misfolding channel opathies is based on pharmacological chaperones. These molecules stabilize protein correct folding, limit premature ER-associated degradation and promote protein delivery to the cell surface. Ivacaftor and lumacaftor, correctors of specific CFTR folding defective mutants, have been successfully used to rescue CFTR dysfunction in patients affected by cystic fibrosis (Mall and Galietta, 2015). Different proof-of-principle strategies have been tested to restore *in vitro* functioning Nav channels in GEFS+, such as the incubation of mutant sodium channels at low temperature, the interactions with different co-expressed proteins (such as β1 subunit, calmodulin, ankyrin G), the exposure to sodium channel blockers such as phenytoin (Rusconi et al., 2007; Cestèle et al., 2013; Bechi et al., 2015). Typical pharmacological chaperones are reversible high affinity ion channels inhibitors, so their application in rescuing misfolded mutants might be limited by the possible block of the rescued channels correctly inserted in the membrane. To overcome these drawbacks in GEFS+, beta scorpion toxins peptidic derivatives were recently engineered to bind Nav channels with high affinity and to target the ER, and appeared more effective pharmacological chaperones than phenytoin *in vitro* (Bechi et al., 2015).

### Voltage Sensor Domain (VSD) Modulators

Voltage sensitivity is provided in voltage gated ion channels by voltage sensor domains, that allow voltage-induced conformational changes leading to channel opening through the interaction of highly conserved charged residues. Most drugs targeting ion channels bind their central alpha pore. Interestingly, disruption of VSDs, mainly due to mutations of arginine residues has been shown to generate gating pore currents that underlie different channelopathies, including hypoPP, peripheral nerve hyperexcitability, epilepsy and arrhythmias (Moreau et al., 2014; Miceli et al., 2015a). Thus, compounds modulating VSDs could be used as research tools and could represent a very promising pharmacological strategy for many pathologies and biophysical defects. Examples of existing VSD modulators are compounds blocking selectively neuronal Na_v_1.1/1.3/1.7 channels, specific small molecule openers or blockers of K_v_7.2 channel (Peretz et al., 2010; McCormack et al., 2013), or guanidinium derivatives blocking the gating currents from mutant Nav1.4 channels (Sokolov et al., 2010). Recently, the small molecule ztz240, selected after a wide and sophisticated screening, has been shown to open K_v_7.2 channels by binding to the F137 residue in the gating charge pathway, and be valuable in the treatment of epilepsy (Kornilov et al., 2013; Li et al., 2013). In addition, gating modifier toxins that target and stabilize the VSD in the resting or activated state, can be useful research tools for structure-function investigations (Moreau et al., 2014; Miceli et al., 2015a).

### Pharmacogenetics

In some channelopathies, the observation that the drug sensitivity of an ion channel *in vitro* depends on the specific biophysical defects carried by single disease-associated ion channel mutations provided the basis for the development of a pharmacogenetic approach. In particular, studies on SCMs demonstrated that sodium channel mutations with a negative shift of the voltage dependence of inactivation showed increased sensitivity to mexiletine, and the corresponding myotonic carriers responded well to this drug. Conversely, mutations that right shifted the inactivation voltage dependence were less sensitive to mexiletine and were preferentially blocked by flecainide (Desaphy et al., 2001, 2004). In the respective mutation carriers, flecainide exhibited greater benefit than mexiletine (Desaphy et al., 2013b). In addition, mutations located close to the binding site for mexiletine can modify sensitivity to the drug (Takahashi and Cannon, 2001). These evidences support the idea that the pharmacological characterization of mutant channels *in vitro* is fundamental to predict drug response *in vivo* and address the carrier of a specific mutation toward the appropriate therapy.

### Marketed Drugs Repurposing

One recent direction of pharmacological research is based on repurposing of existing drugs, that represents a time- and cost-sparing strategy. Nav channel blockers that are already on the market for another indication and can be selected as an alternative to mexiletine to treat neuromuscular channelopathies or cerebellar ataxias represent an example (Desaphy et al., 2014; Matthews and Hanna, 2014; Romano et al., 2015). Importantly, the repurposing of marketed drugs will avoid the need of trials on safety, and, if positive results are obtained in cell and animal models, these drugs will be entered in a phase II clinical trials.

Among novel strategies to discover ion channels selective ligands, it is worth mentioning the search of pharmacovigilance databases to pick commercial drugs inducing a side effect that is endorsed to an ion channel block. Recently this strategy has been used to monitor drug-induced atrophy that *in vitro* resulted from KATP channel block using the Food and Drug Administration-Adverse Effects Reporting System (FDA-AERS) database (Mele et al., 2014). This database is a passive surveillance system that collects spontaneous reports of adverse events regarding any US licensed drugs from providers, health care workers, and the public. Although AERS cannot usually prove causal associations between drugs and adverse events, it can help to obtain helpful information on drug activity that can be validated *in vitro*.

### Novel Targets

Improved understanding of the disease pathways leads to the selection of alternative drug targets apart from the causative ion channel. Indeed, many disorders reflect dysfunctions in regulatory proteins that may alter ion channel biophysical properties, synthesis, membrane trafficking and/or post-translational modifications and/or can modulate the phenotypic variability observed in affected families. These proteins are emerging as attractive opportunities for drug development (Bechi et al., 2015; Brignone et al., 2015). Pharmacological correction of an ion channel defect by targeting an alternative ion channel that may compensate for the primary dysfunction is considered another possible approach to treat channelopathies. Recently, activators of the epithelial Ca^2+^-activated Cl^-^ channels TMEM16A, also known as anoctamin-1 (ANO1), proved useful in reducing sustained inflammation in cystic fibrosis lung disease (Mall and Galietta, 2015). SK channel activators fulfilled this scope in episodic and progressive ataxias (Walter et al., 2006; Romano et al., 2015). Novel ion channels classes, besides classical voltage-gated channels, have emerged as having therapeutic potential, due to their wide distribution and implication in the pathophysiology of several disorders. TRPV channels modulators, for instance, are being developed in inflammatory and neuropathic pain, migraine, focal and segmental glomerulosclerosis, bone abnormalities and other disorders (Kaneko and Szallasi, 2014). Recently, the much-needed molecular identity of volume-regulated anion channel (VRAC) composed of LRRC8 heteromers has been disclosed (Pedersen et al., 2016). Given the pivotal role played in cell volume regulation in all cell types and evidences suggesting the involvement in several pathophysiological processes, VRAC would represent a new promising target in diseases such as cardiac hypertrophy, megalencephalic leukoencephalopathy with subcortical cysts, cerebral ischemia and cancer (Yamamoto et al., 2011; Brignone et al., 2015; Pedersen et al., 2016). In addition, ivabradine, the first hyperpolarization-activated cyclic nucleotide-gated (HCN) channel inhibitor, has been clinically approved for the treatment of chronic stable angina pectoris and heart failure (Cao et al., 2015). Moreover, modulators of acid-sensing ion channels (ASIC) activity are potential candidates for new effective analgesic and neuroprotection drugs (Baron and Lingueglia, 2015).

### Cell and Gene Therapy

Finally, the possibility to manipulate the expression of ion channels by viral gene therapy is another strategy exploited in neurological channelopathies such as pain, epilepsy and muscular dystrophy, where a high percentage of non-responders to traditional pharmacotherapy have been encountered. This challenging approach ranges from the up- or down-regulation of ion channels that control excitability endogenously, to optogenetics and chemogenetics (Snowball and Schorge, 2015).

As additional ion channels are identified and characterized, new channelopathies emerge that would deserve precision medicine. The key to success in the clinical management of ion channelopathies will be the discovery of more potent and selective ion channel modulators to improve efficacy and safety on a larger clinical scale with respect to existing treatment options.

## Author Contributions

PI, DC, and J-FD conceived, coordinated and wrote the paper. AL and GC reviewed kidney channelopathies; MB and AM reviewed skeletal muscle channelopathies; SP, CC, and DT reviewed endocrine channelopathies; J-FD reviewed heart and PNS channelopathies; PI and AG reviewed CNS channelopathies; PI and GC made figures and table; AD edited the paper. All authors approved the final version of the manuscript.

## Conflict of Interest Statement

The authors declare that the research was conducted in the absence of any commercial or financial relationships that could be construed as a potential conflict of interest.
